# Genome-Wide Association Study of Nucleotide Variants Associated with Resistance to Nine Antimicrobials in *Mycoplasma bovis*

**DOI:** 10.3390/microorganisms10071366

**Published:** 2022-07-06

**Authors:** Matthew Waldner, Andrea Kinnear, Elhem Yacoub, Tim McAllister, Karen Register, Changxi Li, Murray Jelinski

**Affiliations:** 1 Western College of Veterinary Medicine, University of Saskatchewan, Saskatoon, SK S7N 5B4, Canada; matthew.waldner@usask.ca (M.W.); andrea.kinnear@usask.ca (A.K.); elhem.yacoub@usask.ca (E.Y.); 2 Lethbridge Research and Development Centre, Agriculture and Agri-Food Canada, Lethbridge, AB T1J 4B1, Canada; tim.mcallister@canada.ca; 3 Ruminant Diseases and Immunology Research Unit, USDA/Agricultural Research Service/National Animal Disease Center, Ames, IA 50010, USA; karen.register@usda.gov; 4 Lacombe Research and Development Centre, Agriculture and Agri-Food Canada, Lacombe, AB T4L 1W1, Canada; changxi.li@agr.gc.ca; 5 Department of Agriculture, Food and Nutritional Science, University of Alberta, Edmonton, AB T6G 2P5, Canada

**Keywords:** *Mycoplasma bovis*, genome-wide association study, antimicrobial resistance, fluoroquinolone, tetracycline, phenicol, macrolide

## Abstract

Antimicrobial resistance (AMR) studies of *Mycoplasma bovis* have generally focused on specific loci versus using a genome-wide association study (GWAS) approach. A GWAS approach, using two different models, was applied to 194 *Mycoplasma bovis* genomes. Both a fixed effects linear model (FEM) and a linear mixed model (LMM) identified associations between nucleotide variants (NVs) and antimicrobial susceptibility testing (AST) phenotypes. The AMR phenotypes represented fluoroquinolones, tetracyclines, phenicols, and macrolides. Both models identified known and novel NVs associated (Bonferroni adjusted *p* < 0.05) with AMR. Fluoroquinolone resistance was associated with multiple NVs, including previously identified mutations in gyrA and parC. NVs in the 30S ribosomal protein 16S were associated with tetracycline resistance, whereas NVs in 5S rRNA, 23S rRNA, and 50S ribosomal proteins were associated with phenicol and macrolide resistance. For all antimicrobial classes, resistance was associated with NVs in genes coding for ABC transporters and other membrane proteins, tRNA-ligases, peptidases, and transposases, suggesting a NV-based multifactorial model of AMR in *M. bovis*. This study was the largest collection of North American *M. bovis* isolates used with a GWAS for the sole purpose of identifying novel and non-antimicrobial-target NVs associated with AMR.

## 1. Introduction

*Mycoplasma bovis* is associated with a variety of cattle diseases having a range of clinical manifestations. In feedlot cattle, *M. bovis* is commonly associated with bovine respiratory disease (BRD) and chronic pneumonia and polyarthritis syndrome (CPPS) [1,2,3,4]. The lack of clinically efficacious vaccines against *M. bovis* has resulted in antimicrobials being administered for the prevention, metaphylaxis, and treatment of mycoplasmosis in feedlot cattle [5]. This extensive use of antimicrobials, however, has potentially contributed to increasing levels of antimicrobial resistance (AMR) in *M. bovis* isolates worldwide [6,7,8,9,10,11,12,13,14,15]. Furthermore, AMR in *M. bovis* can be attributed, in part, to the lack of a cell wall, making it innately resistant to beta-lactams, sulfonamides, trimethoprim, polymyxins, and nalidixic acid [16,17].

Research of AMR determinants of *M. bovis* has primarily focused on antimicrobial target-site modifications (‘hot spots’); specifically, single nucleotide polymorphisms (SNPs) in the genes coding for antimicrobial targets. Macrolide and phenicol resistances are primarily associated with mutations within domains II and IV of the 23S component of the 50S ribosomal subunit, respectively [7,18,19]. Tetracycline resistance is linked to mutations in the 16S component of the 30S subunit [19,20], while mutations in the *gyrA* and *parC* genes reduce the binding affinity of fluoroquinolones [21,22,23,24]. 

Although the aforementioned SNPs have a role in AMR, there is a lack of concordance between in vitro antimicrobial susceptibility testing (AST) results and known SNPs. Khalil et al. surmised that *M. bovis* may have resistance mechanisms other than SNPs, such as efflux pumps that contribute to fluoroquinolone resistance [22]. Similarly, Calcutt et al. noted the need for more research into mechanisms of resistance other than antimicrobial target-site modification [25]. One such method to identify possible mechanisms of resistance is the use of genome-wide association studies (GWAS). GWAS apply a statistical model to associate a set of phenotypic traits with a set of genetic variations, most commonly SNPs, across a set of genomes. 

Only a few GWAS studies have been conducted to characterize AMR in *M. bovis* [26,27], which may be related to the technical expertise required in conducting such studies. Furthermore, a limitation of GWAS is the occurrence of false positive associations between the traits and genetic variants, commonly referred to as “*p*-value inflation” [28]. In bacteria, *p*-value inflation is often caused by a lack of statistical power owing to linkage disequilibrium in clonal populations as well as limited recombinations [28,29,30]. Other causes for false positives include polygenic inheritance for a trait and variant penetrance [31]. Although sample size requirements may differ depending on the variation of phenotypic traits, the number of DNA markers analyzed, and the purpose of the study, there is an underlying convention of having a minimum sample size of 100 bacterial isolates [32,33,34]. Bokma et al. performed a combination GWAS and manual study of 95 *M. bovis* genomes to identify gene targets associated with AMR [27]. This analysis identified known NVs as well as novel variants associated with resistance to fluoroquinolones, macrolides, tetracyclines, and aminoglycosides. However, the GWAS may have been constrained by both the number of isolates and skewed minimum inhibitory concentrations (MIC). Ledger et al. performed a GWAS on two *M. bovis* isolates to identify AMR traits associated with SNPs, multiple nucleotide polymorphisms (MNPs), as well as insertions and deletions (indels) [26]. They identified 77 genes associated with AMR across six different functional groups: topoisomerases, methyltransferases, 30S ribosomal proteins, 50S ribosomal proteins, tRNA ligases, and ABC transporters. 

The objective of this study was to use GWAS to associate (*p* < 0.05) NVs with *M. bovis* AMR phenotypes. The study involved a dataset of 194 *M. bovis* genomes and AMR profiles consisting of MIC values generated from the AST of nine different antimicrobials. Both a fixed effects linear model (FEM) and a linear mixed model (LMM) were used to analyze each dataset, which utilized continuous numeric MICs versus a binary phenotype (susceptible or resistant). Population stratification was considered in the GWAS to account for the compounding effects of isolates obtained from a variety of sources.

## 2. Materials and Methods

### 2.1. Sample Collection, Isolation, DNA Extraction, and Sequencing

*Mycoplasma bovis* isolates (*n* = 194) were acquired from North American feedlot cattle (*n* = 115), farmed bison (*n* = 77), white-tail deer (*n* = 1), and mule deer (*n* = 1). Isolates originated from animals of varying health status (healthy *n* = 39, sick with pneumonia presentation *n* = 17, dead *n* = 134, unknown *n* = four) and derived from different anatomical locations (nasopharynges *n* = 57, lungs *n* = 88, stifle joints *n* = 38, unknown *n* = 11), and over a range of years (2006 to 2018). Deep nasopharyngeal (DNP) swabs were obtained from cattle (*n* = 39) and bison (*n* = 18), while lung and joint samples were obtained at the time of postmortem examination from animals having gross pathological findings consistent with mycoplasmosis (cattle *n* = 76, bison *n* = 48, white-tail deer *n* = one, mule deer *n* = one). The methods for culturing, isolation, DNA extraction, identification, and whole genome sequencing have been previously described [6,7].

### 2.2. Genome Assembly, Quality Control, and Data Preprocessing

Data-preprocessing required each isolate to be indexed to a BAM file, consensus sequence FASTA file, and variant call file (VCF). Trimming of the MiSeq paired-reads was performed in Trimmomatic v0.39 [35] with the following settings: sliding window:5:15, leading:5, trailing:5, and minlen:75. SAM alignment files were created for each read set by aligning the trimmed paired-reads to the *M. bovis* PG45 reference genome (CP002188.1/NC_014760) using BWA mem v0.7.17-r1188 [36]. These SAM files were then converted to BAM, sorted, and indexed with Samtools [37]. Bcftools mpileup and vcf2fq generated the consensus FASTA sequence for each sequenced isolate [37]. All whole genome sequences (WGS) had an average coverage of ≥30X. VCF files were created by inputting the indexed BAM file groups from the assembly stage into freebayes [38], with settings ploidy 1 and strict-vcf. Bcftools was run three times on the resultant VCFs with the following settings: annotate -x ‘FORMAT’, norm -m -, and +missing2ref to remove format tags, normalize multiallelic records into biallelic records, and fill the missing alleles with the reference allele, respectively. Raw reads for each of the genomes have been made available from the Sequence Read Archive (SRA) under BioProject accession no. PRJNA642970, PRJNA708306, and PRJNA785928.

### 2.3. Antimicrobial Resistance Phenotypes

AMR phenotypes for each GWAS were determined by AST using a customized Sensititre™ microplate (Trek Diagnostics, Oakwood, GA, USA) comprised of nine antimicrobials commonly used in western Canada: enrofloxacin (ENRO), chlortetracycline (CTET), oxytetracycline (OXY), florfenicol (FFN), tilmicosin (TIL), tildipirosin (TIP), gamithromycin (GAM), tulathromycin (TUL), and tylosin tartrate (TYLT). The AST method has been previously described [6], and was performed through preparation of the following serial two-fold dilutions: ENRO, 0.12–128 µg/mL; TIP, 0.12–128 µg/mL; GAM, 0.25–256 µg/mL; TUL, 0.25–256 µg/mL; TIL, 1–256 µg/mL; TYLT, 1–128 µg/mL; FFN, 0.25–256 µg/mL; OXY, 0.5–256 µg/mL; and CTET, 1–256 µg/mL. A control in the form of penicillin (2–8 µg/mL) was also prepared. Growth was assessed by using the color redox indicator alamarBlue (Invitrogen™, Thermo Fisher Scientific, Waltham, MA, USA) based on a blue-to-pink color change. MICs were added to a phenotype file with the accompanying isolate identifiers.

### 2.4. Genome-Wide Association Study Pipeline

GWAS were implemented using Pyseer [39], a python-based adaptation of the SEER GWAS suite used for microbial GWAS [40]. A fixed effects linear model (FEM) and a linear mixed model (LMM), were run for each of the nine antimicrobials (18 GWAS). Each model identified associations between NVs and AMR phenotypes, while accounting for confounding population structures. 

The GWAS first estimated the population structure for the FEM and LMM. A pairwise distance matrix was created for the FEM using the FASTA of all isolate consensus sequences as input for mash [41]. Nonparametric multidimensional scaling (MDS) was run on the pairwise distance matrix using the scree_plot_pyseer function. Then, based upon a subjective visual examination of the slope of the scree plot, five dimensions were retained during MDS in the FEM. The FEM also required a gene presence/absence table (Rtab file), with the distance file from mash to determine clusters of orthologous groups of proteins (COGs) for Pyseer execution. The COGs acted as the population substructure to account for the confounding population structure of the dataset during the FEM runs. To facilitate the creation of the Rtab file, Prokka software was used for genome annotation through the generation of GFF files for each assembly [42]. Prokka received a FASTA assembly file of each isolate, an annotation FASTA file of the PG45 reference genome from GenomeNet (downloaded 31 August 2021), and the genetic code setting for *M. bovis* (-gcode 4). The Prokka files were then received by the Roary pipeline [42], which annotated the assemblies to calculate the pan genome. For each GWAS, the phenotype file, Rtab file, mash distance matrix, and dimension cut-off of five were passed to Pyseer to determine the COGs and create an MDS decomposition that functions as the accounting factor for the bacterial population structure. The FEM assumed that the nucleotide variant effect and the MDS effect were fixed effects. The LMM required only a similarity or kinship matrix as the pairwise distance matrix in order to correct for population structure [39], as the nucleotide variant effect was a fixed effect and the population structure effect was random. This matrix was calculated using a VCF file and the list of the isolate identifiers using Pyseer’s similarity_pyseer script. A principle component analysis (PCA) of cattle source location, tissue sample location, disease status, isolation year, and host species were performed using Plink 1.9 [43] against the VCF to determine if isolates clustered by these criteria to determine if known variables were having a fixed effect on the dataset that could be accounted for. Further methods to account for fixed effects due to these variables were unnecessary as it was determined that clustering according to these variables did not occur.

The phenotype file, VCF file, and population matrix file (MDS decomposition matrix for FEM and kinship matrix for LMM) were passed to Pyseer for the GWAS runs. Each run was set to exclude allelic frequencies of <0.02 and >0.98, as per the Pyseer tutorial. The GWAS functioned by applying MDS to the population matrix, projecting the matrix into a reduced number of dimensions. Linear regression using the relevant model and distance was applied to the dataset to determine the variants’ associations to the phenotypes. The GWAS results files were used to create quantile-quantile (Q-Q) plots to identify possible *p*-value inflation for each GWAS. To ensure significance and quality, each set of results was filtered with a Bonferroni corrected *p*-value threshold of 0.05 and the removal of results that failed a chi-squared test. Bonferroni thresholds were determined by the Pyseer script count_patterns.py using the number of unique variant patterns as the number of multiple tests output from each GWAS run. Residuals were plotted and determined to be normally distributed, satisfying an essential assumption for using LMM.

### 2.5. Visualization and Verification

Plots were visualized using the ggplot2 package in R [44]. Manhattan plots, also referred to as −log10(*p*-value) genome-wide association plots, relate the *p*-values of significant NVs (*y*-axis) of the GWAS to a genomic map in base pairs (*x*-axis). In this instance, the PG45 reference genome was used to map the location of the NVs on the *x*-axis. Only the variants meeting the Bonferroni-corrected *p*-value threshold were included in each plot. A selection of NVs were manually labelled with the gene in which the NV was identified based on results with the most significant *p*-values per GWAS and/or the gene containing the NV having been identified in previous AMR studies. Where applicable, NVs identified by the GWAS were verified by the scientific literature. Comparison was achieved by aligning *Escherichia coli* genes against the annotated PG45 *M. bovis* genome in Geneious 2020.1.2 5 (https://www.geneious.com; accessed on 14 September 2021) to determine positioning relative to the nucleotide numbering of the PG45 type strain as per the common notation convention for *M. bovis* NVs and amino acids (AAs).

## 3. Results and Discussion

### 3.1. Analysis of the Genome Assembly Quality and Minimum Inhibitory Concentrations

Assembly results for all 194 genomes are presented in Table S1. The number of reads for each isolate ranged from 131,623 to 664,130, with an average of 292,566. Coverage ranged from 30X to 137X, with an average of 64X. Freebayes identified a total of 83,208 NVs (14,492 in coding regions and 68,716 in non-coding regions). PCA analysis of the NVs with sourcing location, host species, isolation year, disease status, or tissue tropism showed no definitive clustering, suggesting no inherent population stratification due to these variables (Figure S1). 

**Figure 1:**
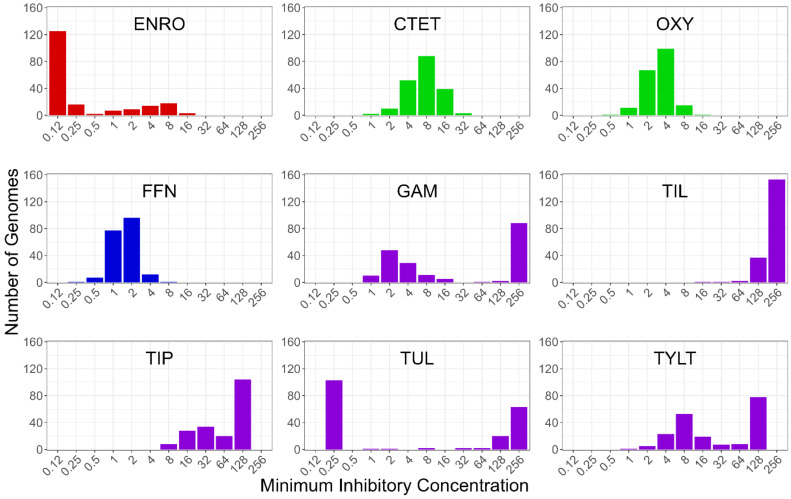
Frequency distributions of *Mycoplasma bovis* isolates (*y*-axis) by minimum inhibitory concentrations (*x*-axis, µg/mL) for each of the nine antimicrobials. Each colour represents an antimicrobial class. Red = fluoroquinolones (ENRO = enrofloxacin); green = tetracyclines (CTET = chlortetracycline, OXY = oxytetracycline); blue = phenicols (FFN = florfenicol), and purple = macrolides (GAM = gamithromycin, TIL = tilmicosin; TIP = tildipirosin, TUL = tulathromycin, TYLT = tylosin tartrate).

Overall, there was wide variation in MIC levels across the *M. bovis* isolates and between antimicrobial classes (Figure 1). Most isolates had low resistance to ENRO with a small number exhibiting resistance up to 16 µg/mL. The CTET and OXY resistant isolates had a similar unimodal distribution at 4–8 and 2–4 µg/mL, respectively. FFN resistance also had a unimodal distribution centered at 1–2 µg/mL. The macrolides showed notable variation in overall MIC profiles even though all five macrolides share a common chemical architecture (Figure S2). Most isolates had high MIC values for TIL and TIP, both of which are derivatives of TYLT, and all three share a 16-membered core structure. Whereas GAM and TUL, which share a 15-membered core structure, had a bimodal MIC distribution, most isolates had low MICs for GAM and TUL. The GWAS models received the MIC data as a continuous variable. Two separate models, FEM and LMM, were used to associate the NVs and MICs. This provided internal confirmation for newly identified NVs. The results of each model are discussed and presented together when they concur, and separately when they do not. Additionally, internal testing of the GWAS pipeline during development showed that the use of a continuous phenotype resulted in lower *p*-value inflation as compared to binary phenotypic data.

### 3.2. Summary Visualization of AMR-Associated Nucleotide Variants within Coding Sequences

Table 1 is a summary of coding sequences that contained significant NVs within gene categories, where significance is defined as having a Bonferroni adjusted *p*-value < 0.05. Results from both the FEM and LMM models have been merged for brevity; however, this information has been summarized in Tables S2–S4, which contain a greater range of descriptive categories. 

**Figure 2:**
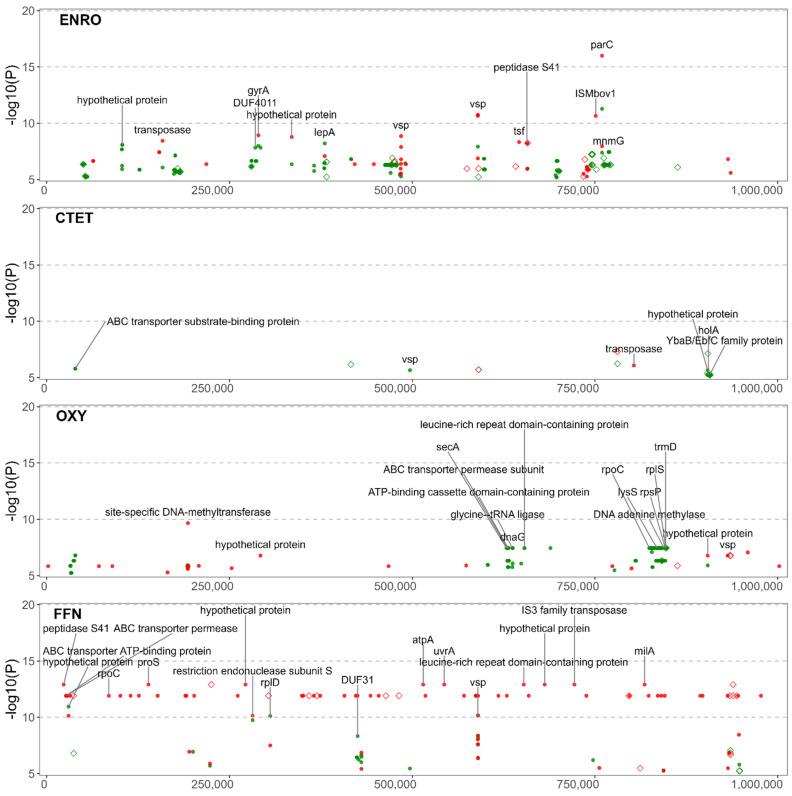
Manhattan plots of variants for enrofloxacin, chlortetracycline, oxytetracycline, and florfenicol resistance. Variants are colour-coded, with fixed effects linear model (green) and linear mixed model (red). The *y*-axis represents the level of statistical significance [−log10 (*p*-value)] with position of the nucleotide variant on the *x*-axis. Significant NVs identified within coding regions are represented with a dot, while significant NVs within non-coding regions are denoted with a hollow diamond.

**Figure 3:**
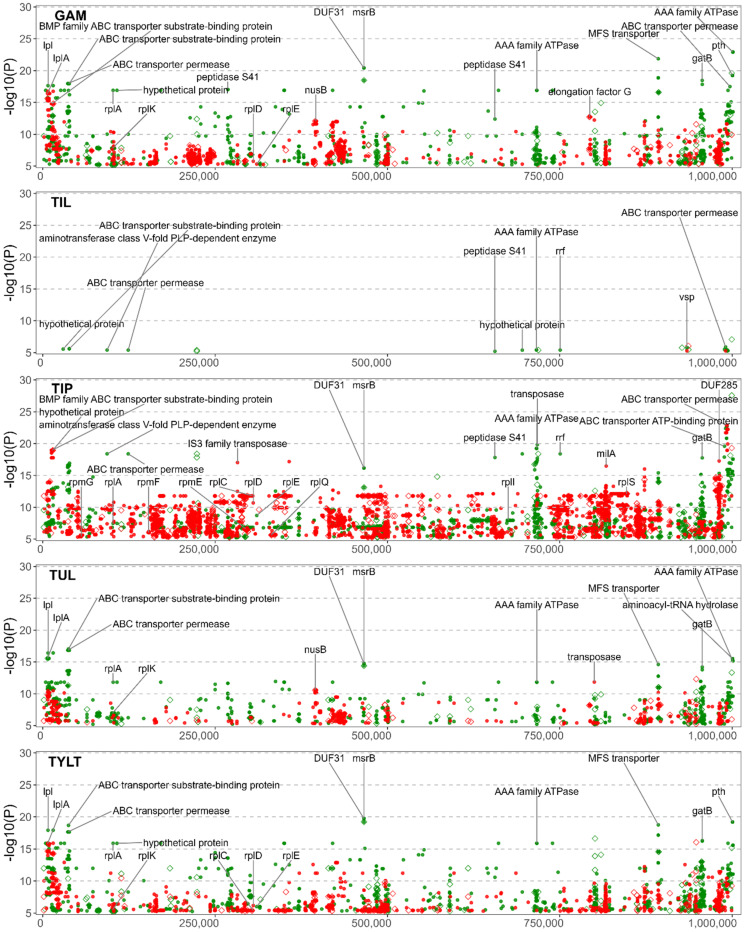
Manhattan plots of variants for gamithromycin, tilmicosin, tildipirosin, tulathromycin, and tylosin tartrate resistance. Variants are colour-coded, with fixed effects linear model (green) and linear mixed model (red). The *y*-axis represents the level of statistical significance [−log10 (*p*-value)] with position of the nucleotide variant on the *x*-axis. Significant NVs identified within coding regions are represented with a dot, while significant NVs within non-coding regions are denoted with a hollow diamond.

Figure 2 is the association of the variants to ENRO, CTET, OXY, and FFN, and Figure 3 provides the association for the five macrolides: GAM. TIL, TIP, TUL and TYLT. The FEM and LMM results are displayed in green and red, respectively. The most significant results for both models have been labelled with the gene of origin. Significant NVs identified within intergenic regions are denoted by a diamond. Only the NVs meeting the corrected *p*-value threshold are included. For each GWAS, the counts of NVs associated with AMR are found in Table S2 and the count of genes that contain significant NVs are found in Table S3. Statistical results for each NV by each GWAS are presented in Table S4. Additional information such as effects on synonymity, acid substitution, and gene name was added through automation.

### 3.3. Investigation into the p-Value Inflation Present within GWAS Results

The inherent caveat to detecting new NV associations with a GWAS is *p*-value inflation, leading to NVs that are falsely associated with the phenotype. Table 2 and Figure S3 show that the ENRO, CTET, OXY, FFN FEM, and TIL FEM GWAS resulted in *p*-value inflation of λ < 1.1, which is acceptable [45]. Values above 1.3 are considered to be high *p*-value inflation resulting in a greater false positive rate [45,46]. The FFN LMM had moderate inflation (λ = 1.17), with the remaining macrolide GWAS having high *p*-value inflation (λ > 1.3). Ideally, the Pyseer pipeline mitigates false positives (Type I error) by defining clusters of orthologous groups (COGs) and using a mixed model approach to control for confounding population effects [29,39]. Potential reasons for the *p*-value inflation include skewed MICs, population stratification, and polygenic inheritance [29,30,31,46,47]. The FFN LMM and macrolide GWAS with the most significant *p*-values, having 10s of variants within a small number of gene families, and having been previously linked to AMR, are discussed below.

### 3.4. Significant NVs Specific to Enrofloxacin Resistance

The NVs with the highest significance to ENRO were identified in the *parC* and *gyrA* genes. Overall, the GWAS identified 468 NVs within and between 68 coding sequences associated with ENRO resistance (Figure 2). NVs were identified within *parC* that included missense mutations resulting in Ser91Ile (*E. coli* Ser80Ile, AGT > ATT) and Asp95Asp (*E. coli* Asp84Asp, GAC > GAT) substitutions. Likewise, within *gyrA* a nonsynonymous mutation encoding for a Ser150Phe (*E. coli* Ser83Phe, TCT > TTT) was prevalent. These mutations have been previously identified as being highly predictive of fluoroquinolone resistance [19,21,22,23]. An NV causing a synonymous mutation in the codon encoding Asp95 of *parC* was also frequently identified. Synonymous mutations are known to affect gene expression through the tendency for certain codons to be used more readily, creating a codon usage bias [48]. This is attributed to the selection of codons for translational efficiency, as has been previously shown for *tuf* genes in *Salmonella* Typhimurium [49].

### 3.5. Significant NVs Specific to Chlortetracycline and Oxytetracycline Resistance

The CTET GWAS identified 242 NVs among 48 coding sequences, while the OXY GWAS identified 50 NVs among nine coding sequences (Figure 2). Unexpectedly, GWAS did not identify known alleles within *rrs1* or *rrs2* associated with tetracycline resistance. Mutations in the 16S rRNA *rrs1* and *rrs2* genes are associated with OXY resistance [19,20]. Nevertheless, multiple NVs associated with OXY AMR were identified in the proteins of the rRNA subunits. The OXY GWAS identified variants in the 30S ribosomal protein S16 (*rpsP* gene) as well as variants in the 50S ribosomal protein L19 (*rplS*). However, the CTET GWAS did not share the same ribosomal protein mutations seen with the OXY resistance. This was unexpected since the mechanisms of action of these two antimicrobials are similar.

### 3.6. Significant NVs Specific to Florfenicol, Gamithromycin, Tilmicosin, Tildipirosin, Tulathromycin, and Tylosin Tartrate Resistance

The phenicol and macrolide GWAS displayed similarity, which was not unexpected since they have similar antimicrobial mechanisms of action. The FFN GWAS identified 108 NVs among 61 coding sequences (Figure 2). The five macrolide GWAS identified NVs and coding sequences associated with AMR as follows: GAM, 3049 NVs, 305 coding sequences; TIL, 75 NVs, 11 coding sequences; TIP, 8847 NVs, 521 coding sequences; TUL, 2003 NVs, 228 coding sequences; and TYLT, 3446 NVs, 368 coding sequences (Figure 3). The increased number of NVs detected in the GAM, TIP, TUL, and TYLT may be attributable to higher *p*-value inflation (λ > 1.3). This is likely due to the location of the NVs within multiple genes for ATP-binding cassette (ABC) transporter proteins, with 10s of NVs within a single gene. The high variability within these genes and the large number of genes coding for transporter proteins plausibly leads to polygenic inheritance as the main contributor to the *p*-value inflation. Conversely, the low number of NVs in the TIL studies can be attributed to most isolates being resistant. *M. bovis* typically has an innate resistance to TIL, with relatively few isolates with a MIC < 256 µg/mL. However, the NVs associated with TIL were also identified in the other macrolide GWAS, and hence are discussed in the multi-drug resistance section below.

NVs associated with phenicol and macrolide resistance were identified in 23S ribosomal RNA (*MBOVPG45_RS01415*) at the well-documented positions of A319980G (*E. coli* A2059G) and A319980C (*E. coli* A2060C) [7,18,19]. Furthermore, FFN and all the macrolide GWAS, except TIL, identified NVs in the 50S ribosomal proteins. Mutations related to FFN resistance were detected in the 50S ribosomal protein L4 (*rplD* gene), while the macrolide GWAS detected NVs in L1 (*rplA*), L3 (*rplC*), L4 (*rplD*), L5 (*rplE*), L9 (*rplI*), L11 (*rplK*), L17 (*rplQ*), L19 (*rplS*), L31 (*rpmE*), L32 (*rpmF*), and L33 (*rpmG*). NVs for the macrolide studies were also detected in genes encoding for proteins of the 30S ribosomal subunit: S2 (*rpsB*), S3 (*rpsC*), S4 (*rpmE*), S6 (*rpsF*), S16 (*rpsP*), S18 (*rpsR*), and S20 (*rpsT*). NVs in 50S ribosomal proteins L1 (*rplA*) and L4 (*rplD*) were commonly identified for the macrolides. However, none of the mutations in L22 (*rplV*) were significant, despite previous reports linking mutations in L4 and L22 to macrolide resistance [7,18,19]. The 5S rRNA subunit of the 50S ribosomal complex was found to contain a non-synonymous mutation (NSM) for several of the macrolides, although with low significance. The lack of identifiable 23S rRNA or 50S rRNA mutations associated with TIL resistance is consistent with previous reports [7,18,19].

With the exception of TIL, a number of macrolide NVs were associated with *msrB*, a gene associated with the repair of oxidative stress in bacteria [50]. Mutations in the MsrB protein have been linked to altered virulence in *Enterococcus faecalis*, with mutants exhibiting greater sensitivity to H_2_O_2_ [50,51]. Although macrolides have been shown to induce oxidative stress associated with toxicity in eukaryotic cells [52,53], this phenomenon has not been well-characterized in bacteria, perhaps because it is not a primary mode of action for macrolides. The lack of research into the effects of *msrB* and oxidative stress in *Mycoplasma* spp. underscores the utility of using GWAS to identify associations between functional genes and AMR. Once identified, these associations can be evaluated for biological plausibility and cross-referenced to other bacteria. 

### 3.7. Variants Associated with Multi-Drug Resistance

NVs within functional genes were associated with multidrug resistance (MDR). These included transporter proteins and membrane proteins; proteins that interact with ribosomes during translation such as tRNA ligases and elongation factors; and proteins that affect mutation, sequence and structure, such as methyltransferases and transposases (Table 1) [26,54,55,56,57,58,59].

All the GWAS identified NVs within the ABC transporter proteins, and the proteins directly interacting with ABC transporter pathways. Genes most prominently identified across all studies included: *MBOVPG45_RS00180*, *MBOVPG45_RS00185*, *MBOVPG45_RS02005*, *MBOVPG45_RS00145*, *MBOVPG45_RS00165*, *MBOVPG45_RS00140*, *MBOVPG45_RS04335*, *MBOVPG45_RS03085*, *MBOVPG45_RS02710*, *MBOVPG45_RS02715*, *MBOVPG45_RS03550*, and *MBOVPG45_RS04315*. These findings are salient because the ABC transporters are a ubiquitous superfamily of membrane and transmembrane proteins that transport substrates such as antimicrobials across the membrane [60]. Transporters acting as antimicrobial efflux pumps have been associated with single and multi-drug resistance in bacteria [61,62]. ABC-type macrolide-specific efflux pumps have been documented in *Mycoplasma pneumoniae* [63], and Ledger et al. suggested that mutations within efflux pumps may also influence AMR in *M. bovis* [26]. Our GWAS identified 19 of the 21 ABC transporters listed by Ledger et al. as containing NVs significant to AMR. The significant NVs linked to TIL resistance were primarily ABC transporters, giving credence to the idea of innate resistance through efflux pumps. Bokma et al. also reported multiple ABC-type macrolide efflux pump genes [27]. 

Multiple NVs were identified in genes coding for other groups of transmembrane and membrane proteins. *MBOVPG45_RS03800*, a gene for the major facilitator superfamily (MFS) of efflux pumps, contained NVs to all of the macrolides investigated with the exception of TIL. MFS transporters play a role in MDR in *E. coli*, *Helicobacter pylori* and other bacterial species [54,64,65,66]. Significant NVs associated with ENRO resistance were within variable surface lipoproteins (Vsps) genes (*MBOVPG45_RS04435*, *MBOVPG45_RS02100*) with a single MNP in both genes containing NVs that cause mutations in 11 AAs. Cell membrane mutations are relevant as they prevent the entry of antimicrobials into the cell, which is a primary mechanism of AMR in all bacteria [67]. Considering that quinolones, such as ENRO, can only cross the cell membrane through porins, mutations in membrane proteins and a reduction in the number of porins via Vsps [67] could contribute to AMR. Mutations in Vsps were also common to FFN and the macrolides, with *MBOVPG45_RS04435* having significant NVs.

ENRO, OXY, FFN and macrolide GWAS, except TIL, all identified NVs within tRNA-ligases including, but not limited to, *argS*, *asnS*, *gltX*, *ileS*, and *lysS*. Also known as aminoacyl-tRNA synthetases, tRNA-ligases transfer single amino acids to tRNAs, enabling peptide synthesis [55]. Mutations in *argS* and *asnS* have been linked to MDR in *E. coli* [68], and mutations in *ileS* confer resistance to mupirocin in *Staphylococcus* spp. [69]. While not a current antimicrobial target, there has been an effort to link mutations in tRNA-ligase to AMR in *M. bovis* [26], which could possibly identify targets for using aminoacyl-tRNA synthetase inhibitors as antimicrobials [55,70]. Ledger et al. listed 22 tRNA ligases, 13 of which we identified as containing NVs to AMR [26].

In addition to the tRNA-ligases, NVs were identified in genes for protein synthesis. These included the aminotransferase class V-fold PLP-dependent enzyme gene *MBOVPG45_RS00395*, whose protein catalyzes the formation of AAs, along with the elongation factors 4 (*lepA*), G (*fusA*), Tu (*tuf*), and Ts (*tsf*) involved in protein synthesis. While there is lack of data regarding NVs in the V-fold PLP-dependent enzyme, mutations within the elongation factors have been associated with AMR in *Mycoplasma* spp., *Pseudomonas aeruginosa*, and *E. coli* [56,57,71,72,73]. 

The LMM GWAS associated GAM, TUL, and TYLT with NVs in the NusB gene *MBOVPG45_RS01740,* which has a role in ribosome biosynthesis by influencing rRNA folding and annealing [74,75]. NVs in *MBOVPG45_RS01740* may result in macrolide resistance by disrupting ribosome biosynthesis [76]. While more research into the possible antimicrobial effects of NusB-NusE dimers is needed, research into identifying antimicrobials that target this interaction are ongoing [77,78].

In all GWAS, except for those conducted on tetracyclines, NVs in genes coding for S41 peptidases (*MBOVPG45_RS00115*, *MBOVPG45_RS01155*, *MBOVPG45_RS01160*, *MBOVPG45_RS02105*, *MBOVPG45_RS02760*, *MBOVPG45_RS02800*, *MBOVPG45_RS02805*) were associated with AMR. The S41 family of peptidases is common to a wide range of organisms. While poorly characterized, these peptidases are believed to have a role in the degradation of incorrectly synthesized proteins and cytoplasmic proteins. The S41 peptidases have been proposed to influence the virulence of *Mycoplasma mycoides capri* by regulating H_2_O_2_ production and modulating cell surface proteins, including IgG-blocking virulence proteins, peptidases, and hypothetical proteins [79,80,81]. 

The tetracycline and macrolide GWAS identified multiple NVs in DNA polymerases, DNA primases, DNA binding proteins, methyltransferases, topoisomerases, and kinases. Although none of these enzymes directly influence the action of tetracyclines, mutations in these genes may contribute to AMR by altering gene expression [82,83,84]. Methylation of the 23S ribosomal RNA at the A2058 (*E. coli* numbering) residue has been shown to directly contribute to macrolide resistance by impairing the binding of macrolides to their active site [58]. Tetracyclines and macrolides were associated with NVs coding for both synonymous mutations (SMs) and NSMs in a total of 24 methyltransferases. These include *rlmB*, *rlmD*, and *MBOVPG45_RS02280*, which Ledger et al. reported to contain NSMs linked to a multi-drug resistant *M. bovis* [26]. RlmB has been speculated to play a role in AMR if its binding activity to 23S rRNA is impeded [26,85]. Excluding previously discussed mutations in topoisomerase genes *parC* and *gyrA* leading to fluroquinolone resistance, NSMs and SMs in *parE, topA*, and a type IIA DNA topoisomerase subunit B (*MBOVPG45_RS04255*) were associated with macrolide resistance. NVs in *parE* and *topA* have been associated with the MDR phenotype in *M. bovis* [26]. Mutations in *topA* have been shown to increase the rate of sequence deletion and duplication, leading to the emergence of AMR genotypes in *E. coli* [86]. 

NVs in transposases or insertion sequences (ISs) were inferred from all analyses except those for TIL. Examples include ISMbov3 family transposases (*MBOVPG45_RS00260*, *MBOVPG45_RS03285*), ISMbov1 family transposases (*MBOVPG45_RS00955*, *MBOVPG45_RS03260*), IS30 family transposases (*MBOVPG45_RS00195*), IS3 family transposases (*MBOVPG45_RS04445*, *MBOVPG45_RS01210*), and other transposases (*MBOVPG45_RS00705*, *MBOVPG45_RS04745*, *MBOVPG45_RS00895*, *MBOVPG45_RS03090*, *MBOVPG45_RS03450*). The contribution of these mobile genetic elements (MGE) in genome diversity and evolution, as well as in modulation of activation and transcription of genes has been described [59]. Horizontal gene transfer (HGT) may drive the development of AMR through the transfer of resistance conferring mutations, as demonstrated by the transfer of ENRO resistance among isolates of *Mycoplasma agalactiae* [87,88]. Little is known, however, about the role of MGE in the emergence of AMR in *M. bovis*. 

It was problematic to interpret the NVs within domains of unknown function (DUFs), hypothetical proteins, and intergenic regions, since few have received functional annotation or been investigated. Genes associated with the DUF31 family of proteins (*MBOVPG45_RS01865*, *MBOVPG45_RS01925*, *MBOVPG45_RS02010*, *MBOVPG45_RS02120*) contained NVs associated with ENRO, FFN and macrolide AMR. DUF31 family proteins are proposed to arise from putative peptidase genes and appear to have a role in the pathogenicity of *Mycoplasma* spp. [81,89,90]. Despite not being protein coding sequences, intergenic or non-coding NVs may have phenotypic consequences. Notably, the GWAS associated intergenic NVs to FFN resistance. These intergenic NVs were positioned nearest to the gene *MBOVPG45_RS03375* encoding an S8 family serine peptidase; *MBOVPG45_RS03860* encoding a hypothetical protein; and *whiA* encoding a probable cell division protein. Likewise, NVs associated with TIL resistance were positioned nearest to *ftsH*, an ATP-dependent zinc metalloprotease, which has a role in membrane proteins, and *MBOVPG45_RS04590*, which is a predicted Vsp. These proteins, however, have not been characterized with respect to AMR. Non-coding NVs such as those mentioned can alter the expression of nearby genes through the alteration of riboswitches, regulatory small RNAs, and transcription promoters, terminators, and regulator binding sites [91]. 

Due to an economized genome, resistance to each antimicrobial has been shown to be associated with mutations in multiple genes rather than possessing specific resistance-conferring genes. Additionally, there exists overlaps in associations of NVs to separate antimicrobials and antimicrobial classes. Therefore, it is likely that a multifactorial model of resistance exists, such that resistance is granted by modifications to antimicrobial targets to prevent antimicrobial binding; modifications to proteins interacting with antimicrobial targets to prevent antimicrobial binding and the repair of cellular damage caused by antimicrobials; and cellular defense mechanisms. Models of resistance such as these have been documented for *E. coli* [92], though in the case of *M. bovis,* resistance originates primarily from NVs in the core genome, as *M. bovis* has not been found to possess novel antimicrobial resistance genes. The results identified by the GWAS have a basis in AMR within *M. bovis* and other bacterial species. Therefore, our results serve as a basis for further research into antimicrobial mechanisms in *M. bovis*.

The study may have benefited from a greater number of isolates, particularly as they relate to the dispersion on AST phenotypes. Most of the isolates were sensitive to ENRO, whereas the AST phenotypes for CTET, OXY and FFN displayed a narrow unimodal distribution. Increasing the number of phenotypes in the extremes, very low and high MIC values may have revealed more associations or increased the statistical significance of some of the associations. The next step would be to validate the associations to substantiate if they are spurious or real. 

## 4. Conclusions

A relatively large body of knowledge exists regarding the specific SNPs involved in antimicrobial target-site modifications, which confer antimicrobial resistance. However, incongruence observed between AMR phenotypes and genotypes suggests that other unidentified mechanisms of resistance exist. Calcutt et al. noted that this is an important knowledge gap and posited, as have others, that transporters may be an important mechanism of resistance [25]. Our findings determined that GWAS is not only an effective method for confirming known target-specific NVs, but has the potential for discovering new NVs and genes associated with AMR. This was particularly true of variants linked to MDR. The GWAS identified proteins that interact with ABC transporters pathways, which are known to be associated with MDR in other bacteria. Mutations were also identified in tRNA-ligases, which have been linked to MDR in *E. coli* and *Staphylococcus* spp. There is also the potential for AMR to be mediated via NVs within S41 peptidases, DNA polymerases and primases, methyltransferases, and kinases, to name but a few. Less well understood is the significance of NVs within domains of unknown functions, hypothetical proteins, and intergenic regions. Although poorly understood, these NVs should not be overlooked, as they may inform AMR as well as transmission and pathogenicity. A multifactorial model of AMR in *M. bovis* likely exists, as modifications to antimicrobial targets, antimicrobial target-interacting proteins, and cellular defense mechanisms such as transporters and Vsps were found to be associated with AMR. Further GWAS utilizing larger and more diverse datasets may uncover additional genetic markers for AMR.

## Figures and Tables

**Table 1:** Summary of coding sequences containing significant NVs within gene categories for each antimicrobial.

Category	ENRO	CTET	OXY	FFN	GAM	TIL	TIP	TUL	TYLT
30S rRNA and proteins	0	0	1	0	1	0	6	1	3
50S rRNA and proteins	0	0	1	1	5	0	11	3	6
ABC transporter	2	1	8	5	24	4	32	25	27
ATPase	1	0	2	1	4	1	9	3	4
Elongation factor	1	0	0	0	1	0	2	1	2
Hypothetical protein	12	1	5	14	68	2	107	45	74
Membrane protein	1	0	0	3	6	0	9	3	8
Methyltransferase	2	0	6	4	8	0	21	6	10
Nuclease	4	0	2	3	12	0	22	9	18
Peptidase	3	0	1	3	13	1	22	12	16
Polymerase	1	2	4	2	4	0	11	2	6
Topoisomerase	2	1	0	1	2	0	6	1	4
Transmembrane protein	2	0	0	0	4	0	9	2	6
Transmem. transport protein	0	0	1	0	3	0	9	3	6
Transposase	5	1	3	3	20	0	31	14	23
tRNA Ligase	2	0	3	2	2	0	13	2	4
Uncharacterized protein	4	0	3	1	14	0	20	10	12
Variable surface lipoproteins	5	1	1	5	16	1	19	14	17

**Table 2:** Genomic inflation factors (λ) for each GWAS, where λ is the ratio of the median of the empirically observed distribution of the test statistic to the expected median.

GWAS Model	ENRO	CTET	OXY	FFN	GAM	TIL	TIP	TUL	TYLT
Fixed Effects Model	1.06	1	1	1	3.1	1	2.85	2.27	2.45
Linear Mixed Model	1	1	1.02	1.17	3.87	1.89	4.84	3.63	2.93

## Data Availability

The raw paired-end read files analyzed in this study are openly available the Sequence Read Archive (SRA) under BioProject accession no. PRJNA642970, PRJNA708306, and PRJNA785928.

## Supplementary Materials

The following supporting information can be downloaded at: https://www.mdpi.com/article/10.3390/microorganisms10071366/s1, Table S1: Assembly metadata of the 194 *Mycoplasma bovis* genomes enrolled in this study. Table S2: Counts of the significant nucleotide variants for each GWAS. Table S3: Counts of the genes containing significant nucleotide variants for each GWAS. Table S4: Results for all performed genome-wide association studies with metadata. Figure S1: PCA analysis of various metadata against the VCF dataset. Figure S2: Minimum inhibitory concentration values for each *Mycoplasma bovis* isolate. Figure S3: Quantile-quantile plots for each genome-wide association study.
