# Supplementary material for: Genome-Wide Association Study of Nucleotide Variants Associated with Resistance to Nine Antimicrobials in Mycoplasma bovis
Source: Microorganisms. 2022 Jul 6;10(7):1366. doi: 10.3390/microorganisms10071366 (PMC9320666; doi:10.3390/microorganisms10071366)
Supplement: Supplementary file 1 [file microorganisms-10-01366-s001.zip › microorganisms-1799817-supplementary/Supplementary_Materials.pdf]

Supplementary Tables:

Table S1: Assembly metadata of the 194 Mycoplasma bovis genomes enrolled in this study. This table contains statistics on the raw reads, reference assemblies, and additional *de novo* assemblies performed using SPAdes with settings as previously described [7].

(The table is present in Table_S1.csv)

Table S2: Counts of the significant nucleotide variants for each GWAS. Each genome-wide association study has the counts of NVs separated by level of significance. The variants have been binned into three groups: variants within coding sequences that cause synonymous mutations (synonymous coding), variants within genes that cause non-synonymous mutations (non-synonymous coding), and non-coding variants.

	Number of Significant Nucleotide Variants by Study (Synonymous Coding / Non-Synonymous Coding / Non-Coding)									
Level of Sig. -log10(*p*-value)	ENRO	FFN	CTET	OXY	CTET	GAM	TIL	TIP	TUL	TYLT
5 - 10	253 / 158 / 53	11 / 23 / 6	28 / 15 / 7	164 / 66 / 12	28 / 15 / 7	1045 / 1040 / 300	29 / 40 / 6	3097 / 2764 / 827	818 / 753 / 171	1317 / 1164 / 388
10 - 15	1 / 2 / 0	11 / 45 / 12	-	-	-	264 / 248 / 41	-	891 / 652 / 226	122 / 95 / 14	244 / 229 / 38
15 - 20	0 / 1 / 0	-	-	-	-	42 / 36 / 5	-	153 / 165 / 14	20 / 9 / 1	29 / 31 / 6
20 - 25	-	-	-	-	-	7 / 3 / 0	-	25 / 32 / 0	-	-
25 - 30	-	-	-	-	-	-	-	0 / 0 / 1	-	-
Total	254 / 161 / 53	22 / 68 / 18	28 / 15 / 7	164 / 66 / 12	28 / 15 / 7	1358 / 1327 / 364	29 / 40 / 6	4166 / 3613 / 1068	960 / 857 / 186	1590 / 1424 / 432

Table S3: Counts of the genes containing significant nucleotide variants for each GWAS. The table contains counts of the number of identified genes, domains, rRNA or other feature containing at least one significant nucleotide variant for each genome-wide association study, separated by significance.

	Number of Coding Sequences Containing Significant Variants Per Antimicrobial								
Level of Sig. -log10(*p*-value)	ENRO	FFN	CTET	OXY	GAM	TIL	TIP	TUL	TYLT
5 - 10	65	10	9	48	208	11	312	180	259
10 - 15	2	51	0	0	65	0	180	39	80
15 - 20	1	0	0	0	28	0	28	9	29
20 - 25	0	0	0	0	4	0	1	0	0
25 - 30	0	0	0	0	0	0	0	0	0
Total	68	61	9	48	305	11	521	228	368

Table S4: Results for all performed genome-wide association studies with metadata. The identity of each study antimicrobial study can be sorted by the “GWAS ID” (F) and “GWAS Type” (G) columns. Information on the gene containing each nucleotide variant and relevant protein product is available in columns A-C, and information on the nucleotide variant and the amino acid mutation it contributes to is available in columns D, E, and H-M. Note that the algorithm used to annotate the amino acid mutations does not take non-coding gene features (such as rRNA) into account and therefore describes non-existent amino acid mutations. Columns N-R contain the statistical information for that nucleotide variant from the genome-wide association study. Columns S-U contain additional categorization information for each nucleotide variant.

(The table is present in Table_S4.csv)

Supplementary Figures:

Figure S1.X: PCA analysis of various metadata against the VCF dataset. Each set of 5 sets of metadata was plotted separately in the subfigures below. Points within the graph represent individual samples that have been coloured according to metadata. Heavy clustering by a specific piece of metadata was not found to be present in the plots.

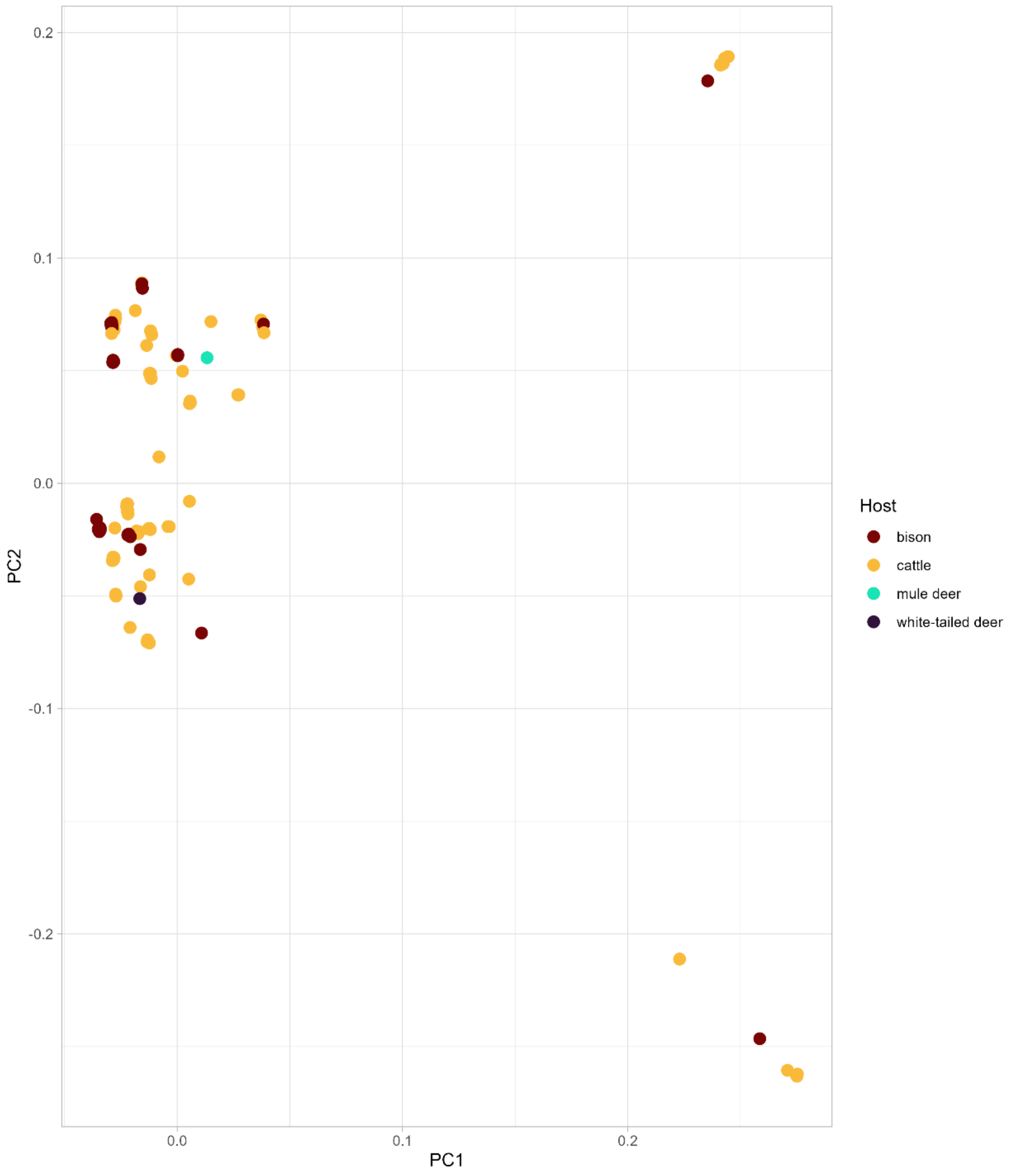

Figure S1.1: PCA plot of the host species against the VCF dataset.

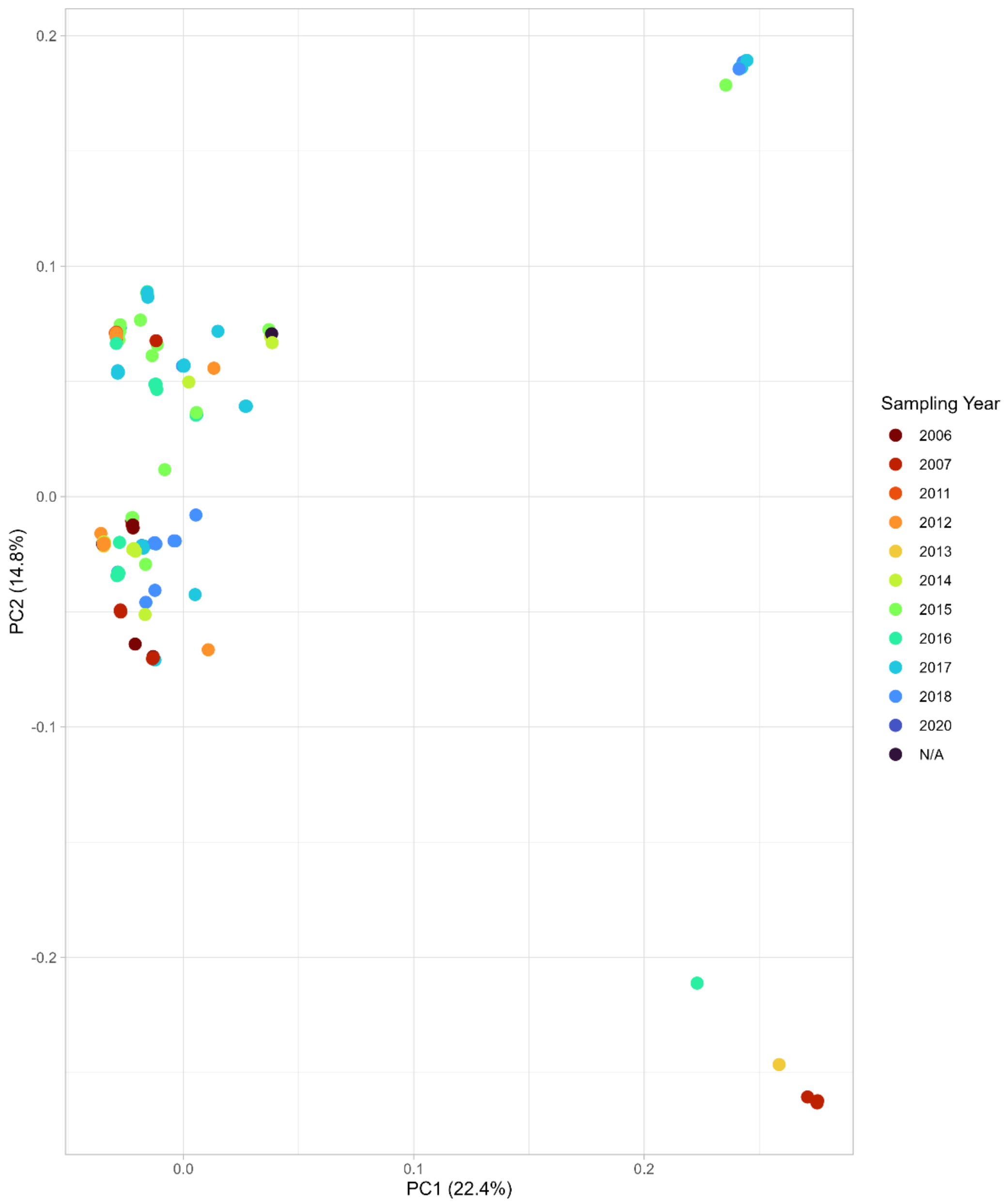

Figure S1.2: PCA plot of the sampling year against the VCF dataset.

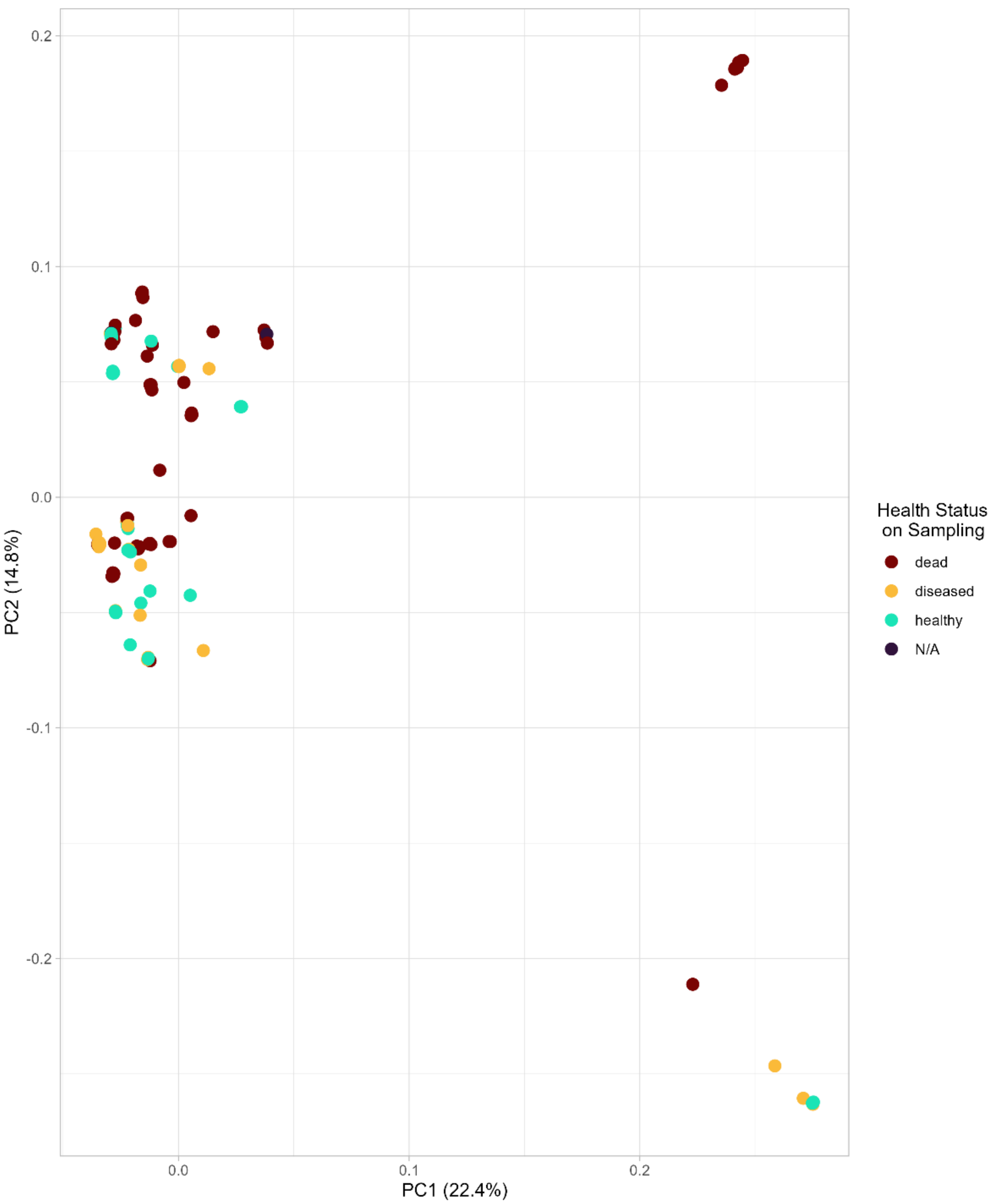

Figure S1.3: PCA plot of animal health status at sampling time against the VCF dataset.

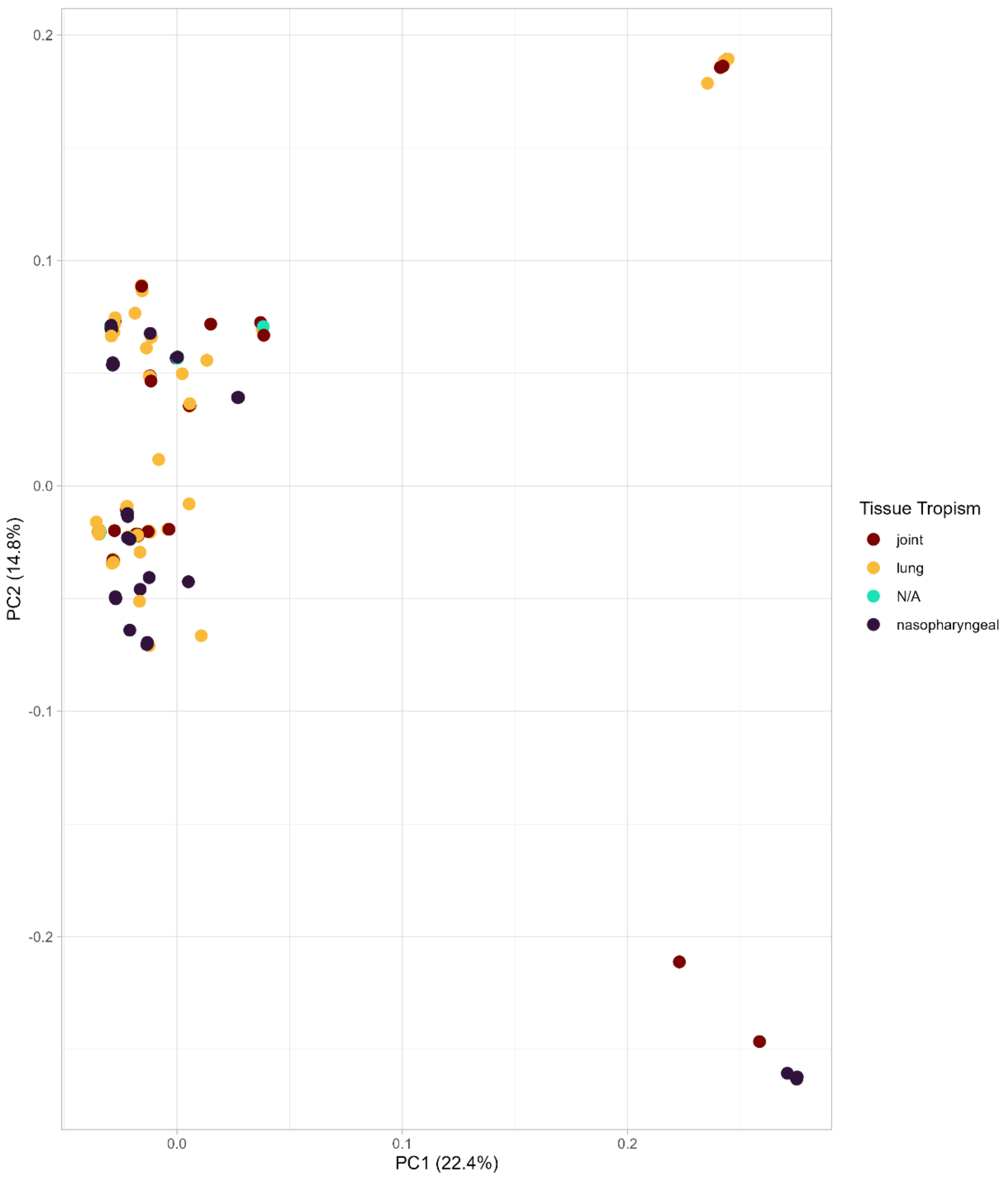

Figure S1.4: PCA plot of sample tissue tropism against the VCF dataset.

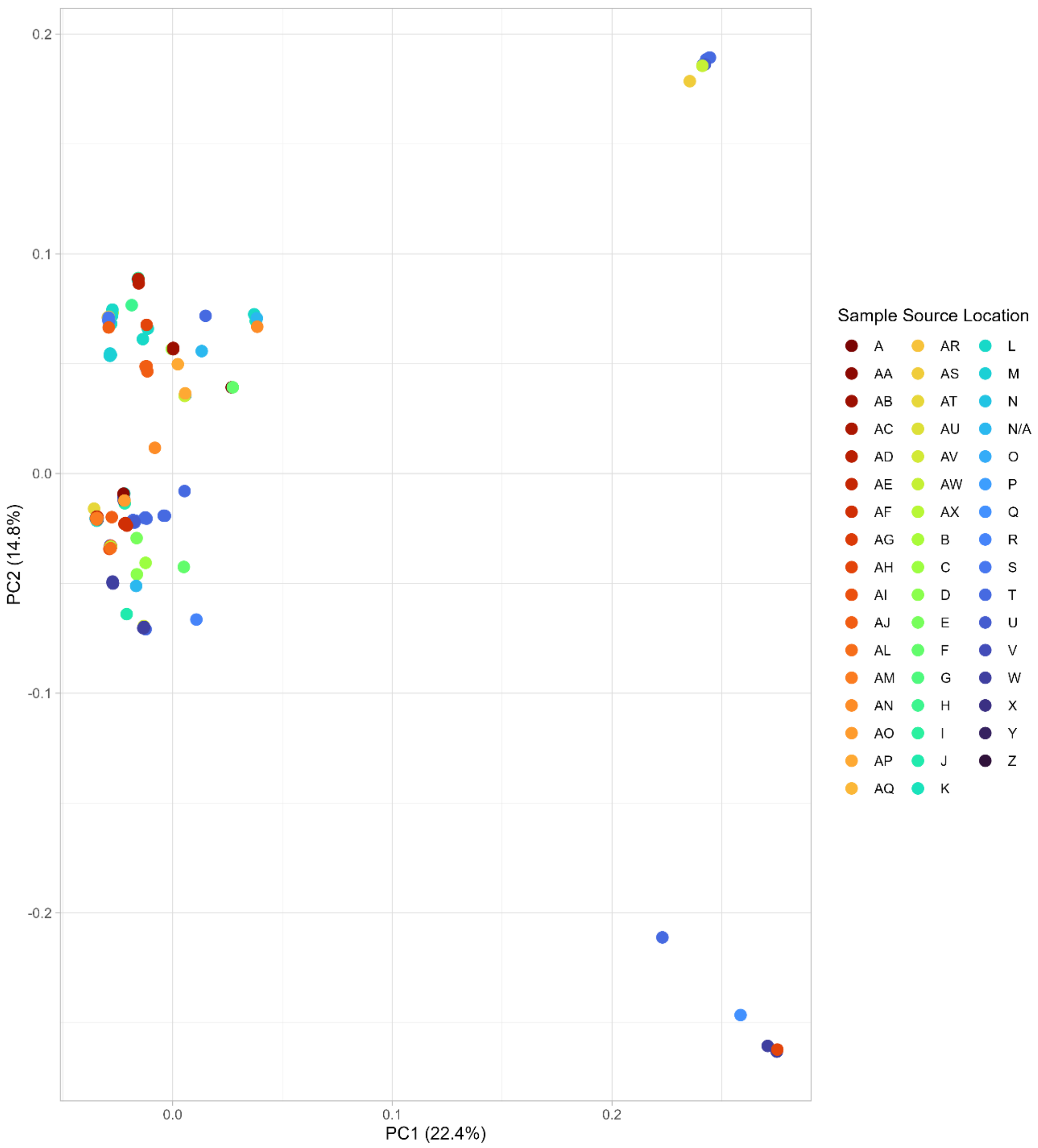

Figure S1.5: PCA plot of cattle source location against the VCF dataset.

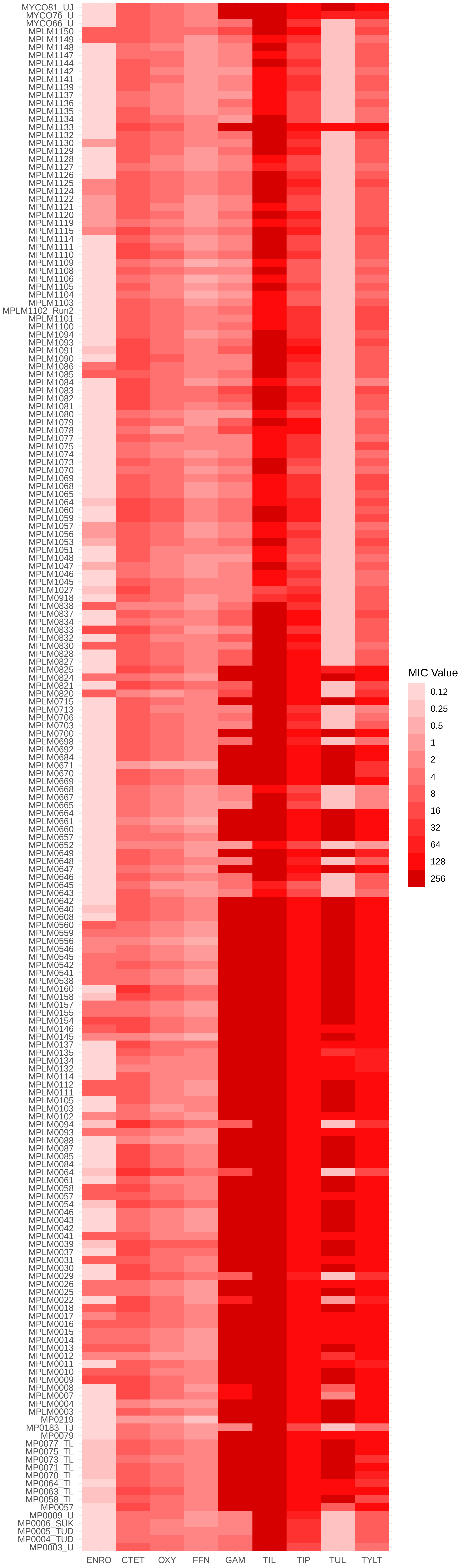
MYCO81_UJ
MYCO76_U
MYCO66_U
MPLM1150
MPLM1149
MPLM1148
MPLM1147
MPLM1144
MPLM1142
MPLM1141
MPLM1139
MPLM1137
MPLM1136
MPLM1135
MPLM1134
MPLM1133
MPLM1132
MPLM1130
MPLM1129
MPLM1128
MPLM1127
MPLM1126
MPLM1125
MPLM1124
MPLM1122
MPLM1121
MPLM1120
MPLM1119
MPLM1115
MPLM1114
MPLM1111
MPLM1110
MPLM1109
MPLM1108
MPLM1106
MPLM1105
MPLM1104
MPLM1103
MPLM1102_Run2
MPLM1101
MPLM1100
MPLM1094
MPLM1093
MPLM1091
MPLM1090
MPLM1086
MPLM1085
MPLM1084
MPLM1083
MPLM1082
MPLM1081
MPLM1080
MPLM1079
MPLM1078
MPLM1077
MPLM1075
MPLM1074
MPLM1073
MPLM1070
MPLM1069
MPLM1068
MPLM1065
MPLM1064
MPLM1060
MPLM1059
MPLM1057
MPLM1056
MPLM1053
MPLM1051
MPLM1048
MPLM1047
MPLM1046
MPLM1045
MPLM1027
MPLM0918
MPLM0838
MPLM0837
MPLM0834
MPLM0833
MPLM0832
MPLM0830
MPLM0828
MPLM0827
MPLM0825
MPLM0824
MPLM0821
MPLM0820
MPLM0715
MPLM0713
MPLM0706
MPLM0703
MPLM0700
MPLM0698
MPLM0692
MPLM0684
MPLM0671
MPLM0670
MPLM0669
MPLM0668
MPLM0667
MPLM0665
MPLM0664
MPLM0661
MPLM0660
MPLM0657
MPLM0652
MPLM0649
MPLM0648
MPLM0647
MPLM0646
MPLM0645
MPLM0643
MPLM0642
MPLM0640
MPLM0608
MPLM0560
MPLM0559
MPLM0556
MPLM0546
MPLM0545
MPLM0542
MPLM0541
MPLM0538
MPLM0160
MPLM0158
MPLM0157
MPLM0155
MPLM0154
MPLM0146
MPLM0145
MPLM0137
MPLM0135
MPLM0134
MPLM0132
MPLM0114
MPLM0112
MPLM0111
MPLM0105
MPLM0103
MPLM0102
MPLM0094
MPLM0093
MPLM0088
MPLM0087
MPLM0085
MPLM0084
MPLM0064
MPLM0061
MPLM0058
MPLM0057
MPLM0054
MPLM0046
MPLM0043
MPLM0042
MPLM0041
MPLM0039
MPLM0037
MPLM0031
MPLM0030
MPLM0029
MPLM0026
MPLM0025
MPLM0022
MPLM0018
MPLM0017
MPLM0016
MPLM0015
MPLM0014
MPLM0013
MPLM0012
MPLM0011
MPLM0010
MPLM0009
MPLM0008
MPLM0007
MPLM0004
MPLM0003
MP0219
MP0183_TJ
MP0079
MP0077_TL
MP0075_TL
MP0073_TL
MP0071_TL
MP0070_TL
MP0064_TL
MP0063_TL
MP0058_TL
MP0057
MP0009_U
MP0006_SUK
MP0005_TUD
MP0004_TUD
MP0003_U
ENRO
CTET
OXY
FFN
GAM
TIL
TIP
TUL
TYLT
MIC Value
0.12
0.25
0.5
1
2
4
8
16
32
64
128
256

Figure S2: Minimum inhibitory concentration values for each *Mycoplasma bovis* isolate. The rows represent each of the 194 isolates, with identifiers for each isolate present on the left side of the heatmap. The columns represent each of the nine antimicrobials and the colour intensity of each cell represents the minimum inhibitory concentration value for that combination of isolate and antimicrobial, as shown in the legend.

Figure S3.X: Quantile-quantile plots for each genome-wide association study. Each point in the plot represent a nucleotide variant. The x-axis describes the expected -log10(p-value) for each variant while the y-axis describes the observed -log10(p-value). P-value inflation is said to occur when the observed -log10(p-value) are higher than the expected -log10(p-value) at -log10(p-value) = 1.

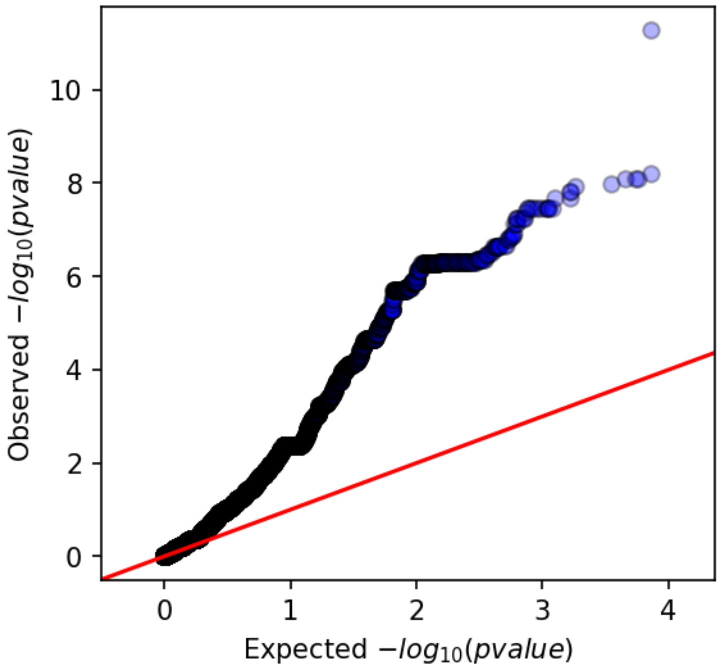

Figure S3.1: Quantile-Quantile Plot for the Enrofloxacin Fixed Effect Model GWAS

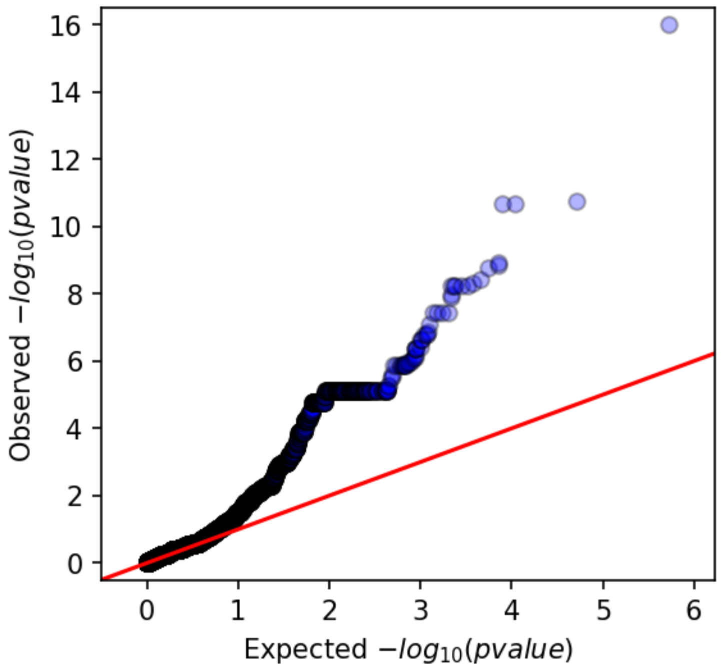

Figure S3.2: Quantile-Quantile Plot for the Enrofloxacin Linear Mixed Models GWAS

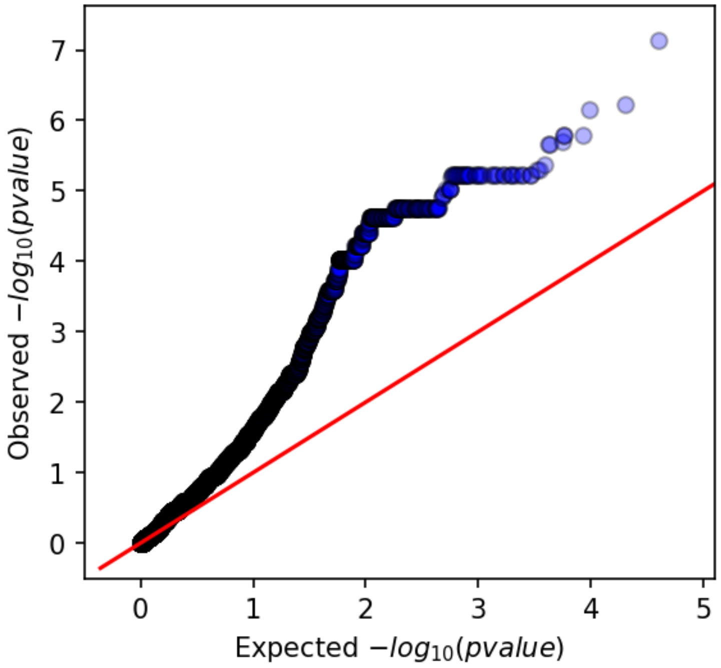

Figure S3.3: Quantile-Quantile Plot for the Chlortetracycline Fixed Effect Model GWAS

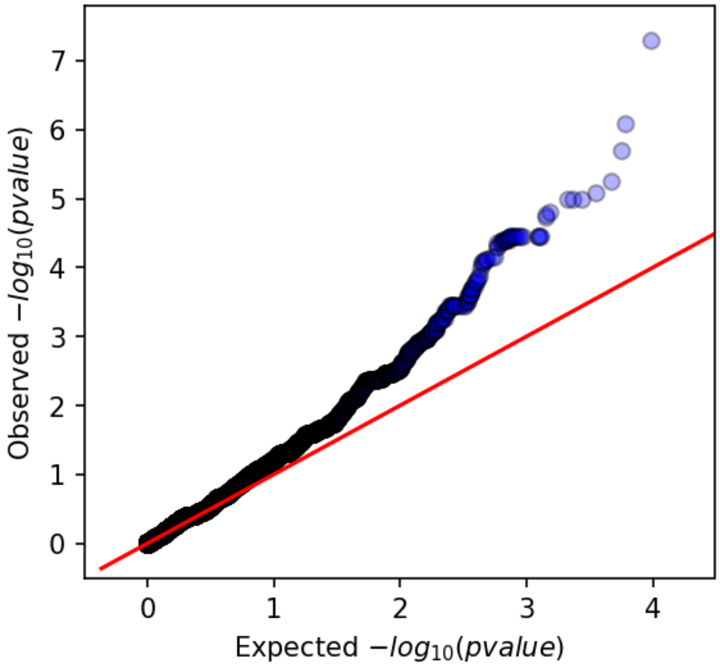

Figure S3.4: Quantile-Quantile Plot for the Chlortetracycline Linear Mixed Models GWAS

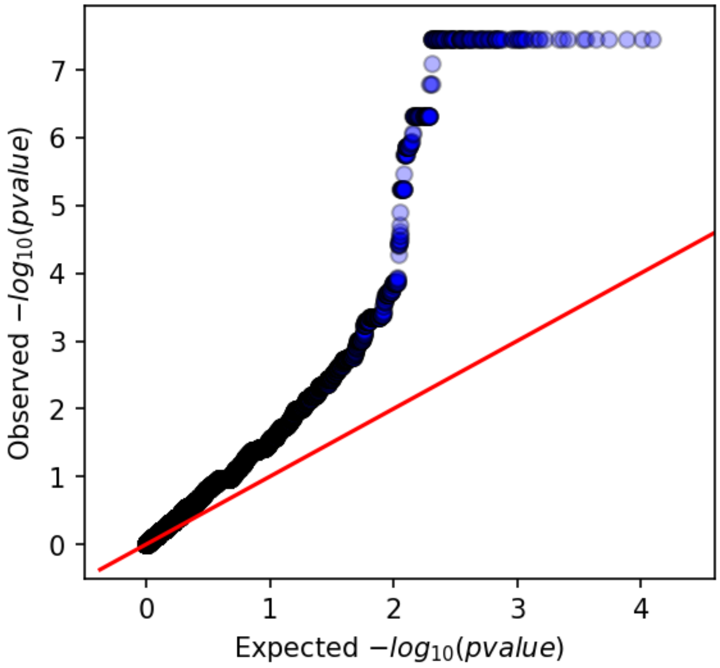

Figure S3.5: Quantile-Quantile Plot for the Oxytetracycline Fixed Effect Model GWAS

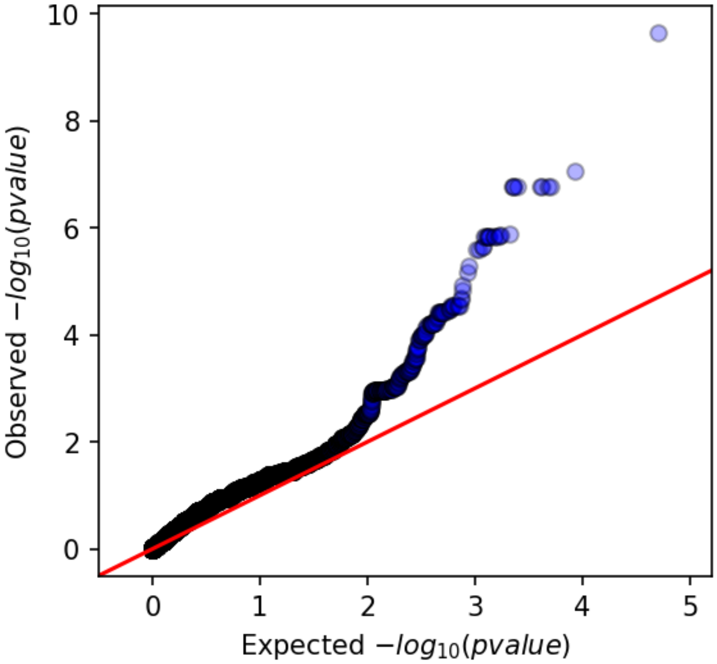

Figure S3.6: Quantile-Quantile Plot for the Oxytetracycline Linear Mixed Models GWAS

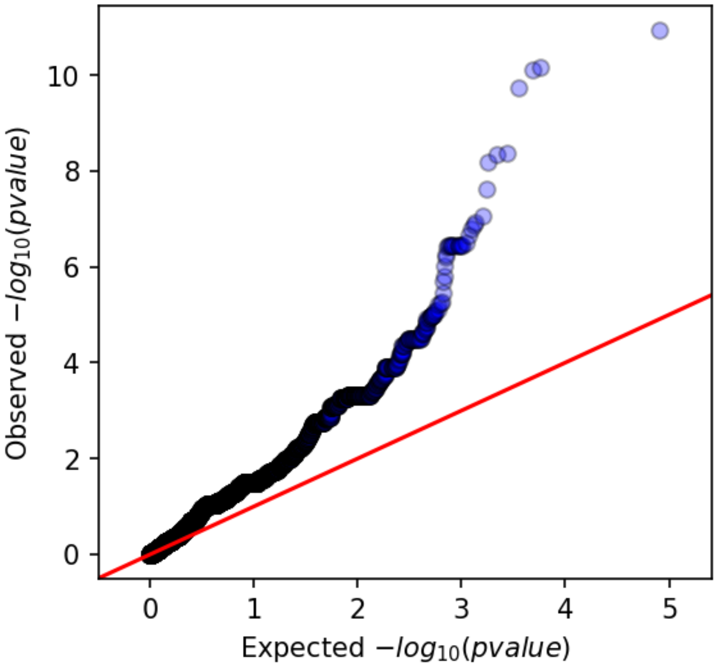

Figure S3.7: Quantile-Quantile Plot for the Florfenicol Fixed Effect Model GWAS

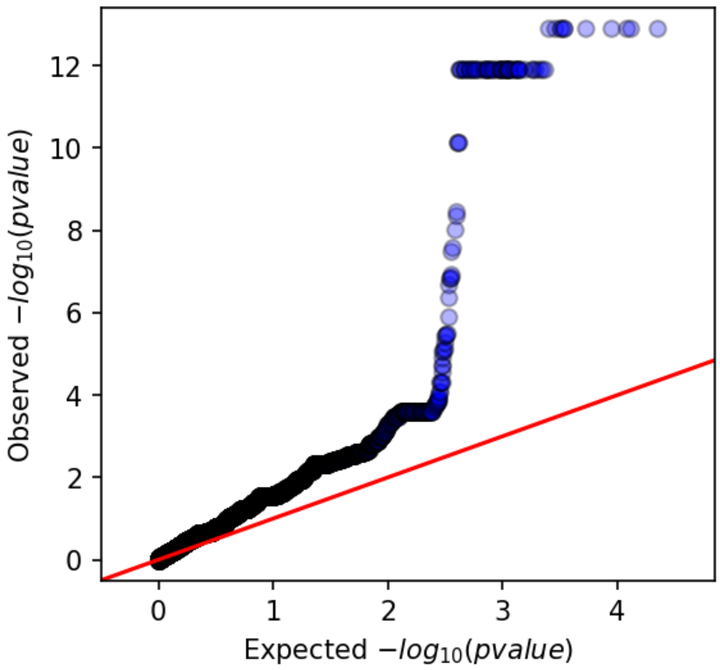

Figure S3.8: Quantile-Quantile Plot for the Florfenicol Linear Mixed Models GWAS

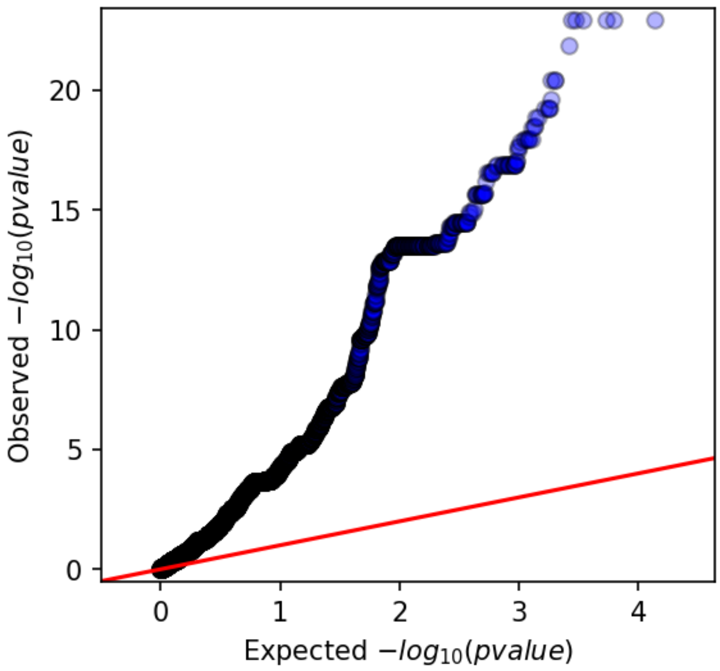

Figure S3.9: Quantile-Quantile Plot for the Gamithromycin Fixed Effect Model GWAS

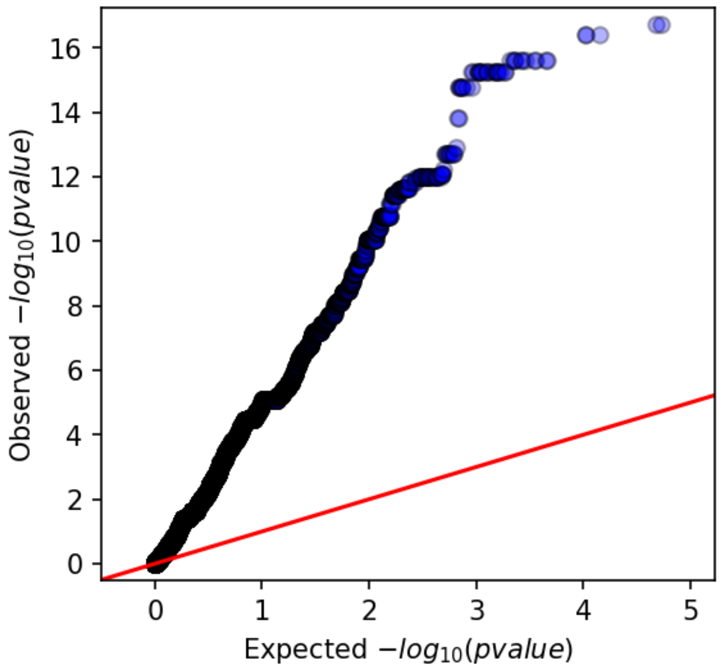

Figure S3.10: Quantile-Quantile Plot for the Gamithromycin Linear Mixed Models GWAS

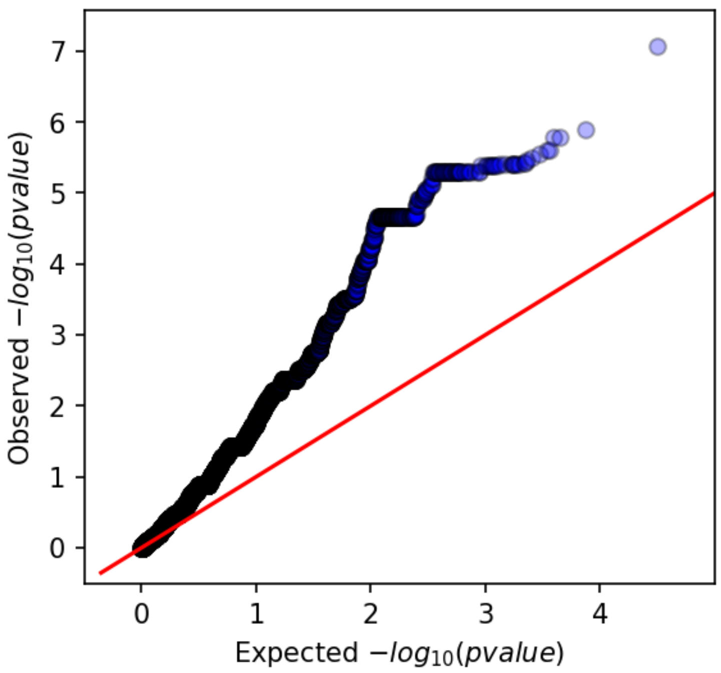

Figure S3.11: Quantile-Quantile Plot for the Tilmicosin Fixed Effect Model GWAS

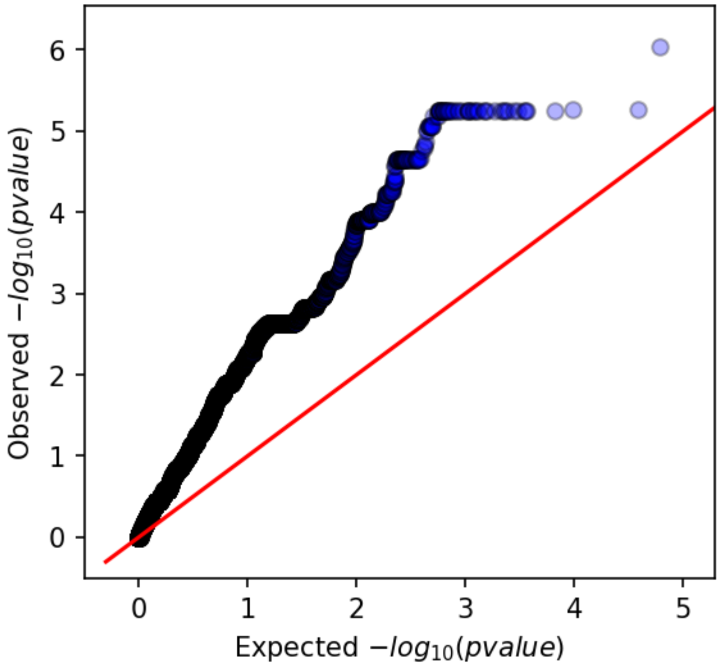

Figure S3.12: Quantile-Quantile Plot for the Tilmicosin Linear Mixed Models GWAS

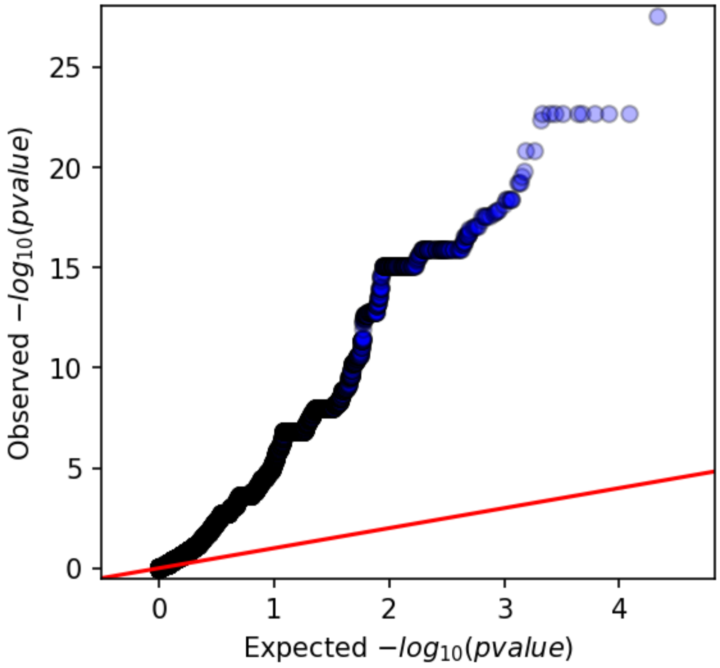

Figure S3.13: Quantile-Quantile Plot for the Tildipirosin Fixed Effect Model GWAS

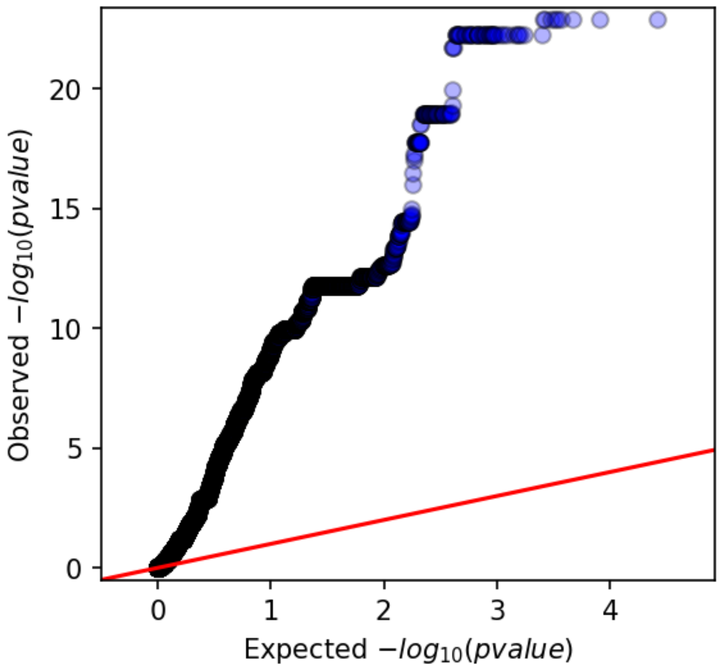

Figure S3.14: Quantile-Quantile Plot for the Tildipirosin Linear Mixed Models GWAS

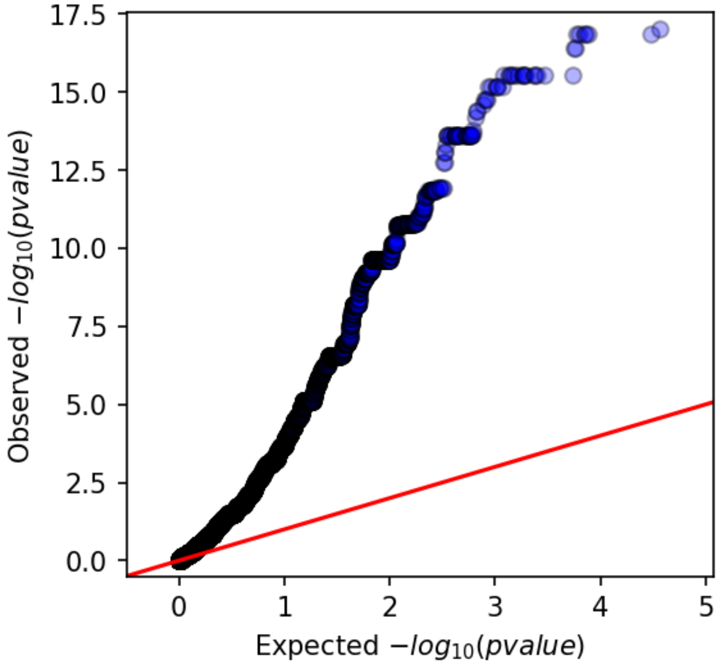

Figure S3.15: Quantile-Quantile Plot for the Tulathromycin Fixed Effect Model GWAS

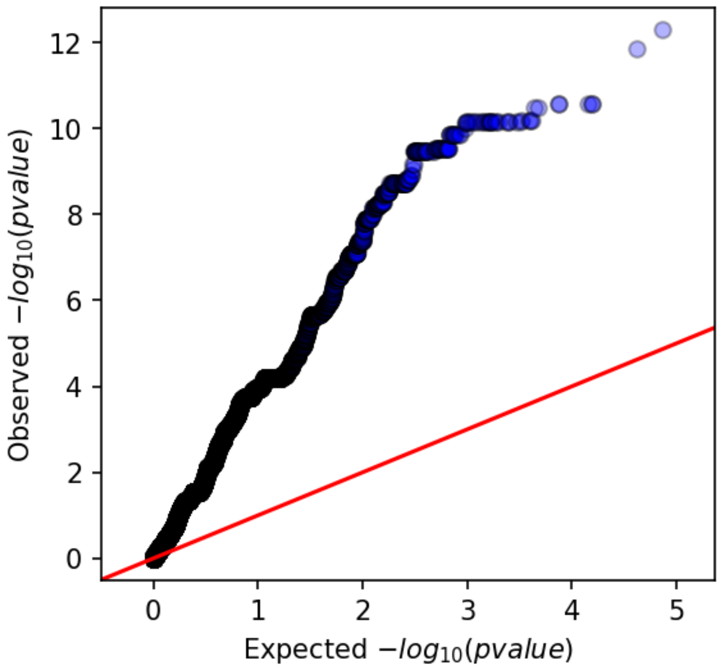

Figure S3.16: Quantile-Quantile Plot for the Tulathromycin Linear Mixed Models GWAS

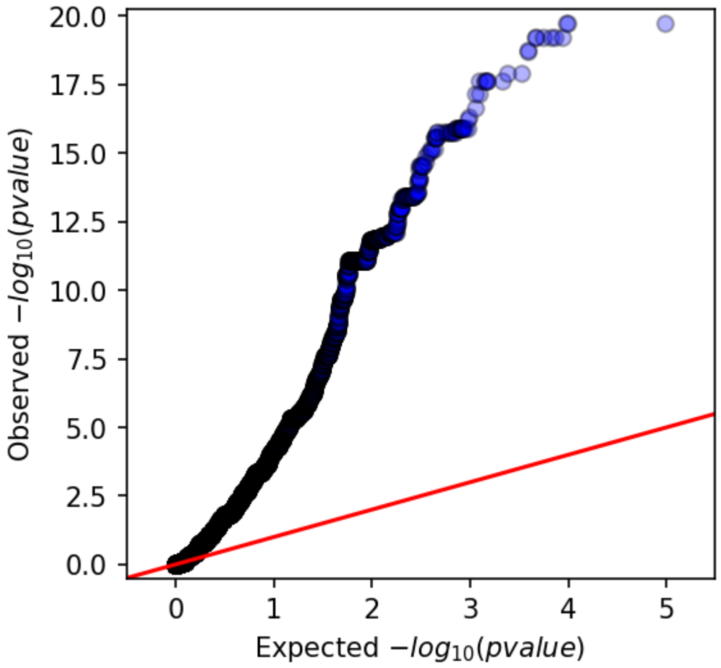

Figure S3.17: Quantile-Quantile Plot for the Tylosin Tartrate Fixed Effect Model GWAS

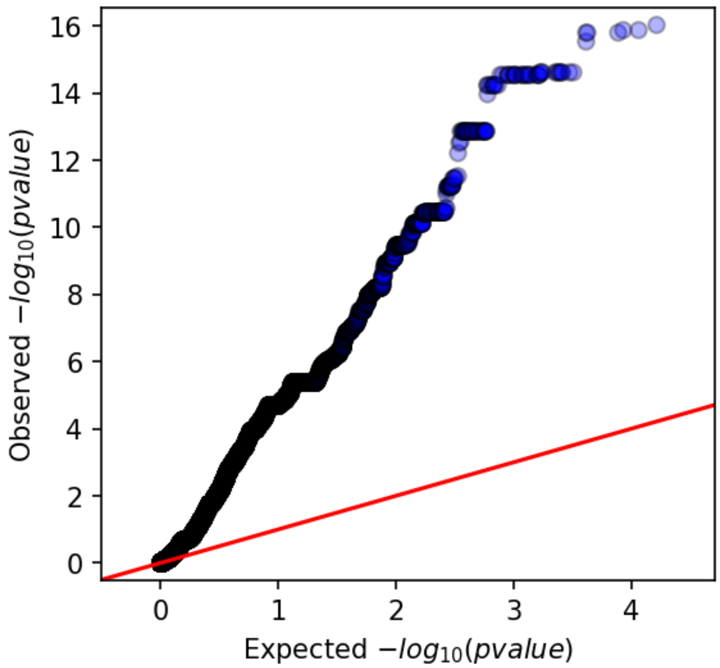

Figure S3.18: Quantile-Quantile Plot for the Tylosin Tartrate Linear Mixed Models GWAS
